# Comparative photon and proton dosimetry for patients with mediastinal lymphoma in the era of Monte Carlo treatment planning and variable relative biological effectiveness

**DOI:** 10.1186/s13014-019-1432-8

**Published:** 2019-12-30

**Authors:** Yolanda D. Tseng, Shadonna M. Maes, Gregory Kicska, Patricia Sponsellor, Erik Traneus, Tony Wong, Robert D. Stewart, Jatinder Saini

**Affiliations:** 1 Department of Radiation Oncology, University of Washington, 1959 NE Pacific Street, Box 356043, Seattle, WA 98195 USA; 2 Seattle Cancer Care Alliance Proton Therapy Center, Seattle, WA USA; 3 Department of Radiology, University of Washington, Seattle, WA USA; 4 RaySearch Laboratories AB, Stockholm, Sweden

## Abstract

**Background:**

Existing pencil beam analytical (PBA) algorithms for proton therapy treatment planning are not ideal for sites with heterogeneous tissue density and do not account for the spatial variations in proton relative biological effectiveness (vRBE). Using a commercially available Monte Carlo (MC) treatment planning system, we compared various dosimetric endpoints between proton PBA, proton MC, and photon treatment plans among patients with mediastinal lymphoma.

**Methods:**

Eight mediastinal lymphoma patients with both free breathing (FB) and deep inspiration breath hold (DIBH) CT simulation scans were analyzed. The original PBA plans were re-calculated with MC. New proton plans that used MC for both optimization and dose calculation with equivalent CTV/ITV coverage were also created. A vRBE model, which uses a published model for DNA double strand break (DSB) induction, was applied on MC plans to study the potential impact of vRBE on cardiac doses. Comparative photon plans were generated on the DIBH scan.

**Results:**

Re-calculation of FB PBA plans with MC demonstrated significant under coverage of the ITV V99 and V95. Target coverage was recovered by re-optimizing the PT plan with MC with minimal change to OAR doses. Compared to photons with DIBH, MC-optimized FB and DIBH proton plans had significantly lower dose to the mean lung, lung V5, breast tissue, and spinal cord for similar target coverage. Even with application of vRBE in the proton plans, the putative increase in RBE at the end of range did not decrease the dosimetric advantages of proton therapy in cardiac substructures.

**Conclusions:**

MC should be used for PT treatment planning of mediastinal lymphoma to ensure adequate coverage of target volumes. Our preliminary data suggests that MC-optimized PT plans have better sparing of the lung and breast tissue compared to photons. Also, the potential for end of range RBE effects are unlikely to be large enough to offset the dosimetric advantages of proton therapy in cardiac substructures for mediastinal targets, although these dosimetric findings require validation with late toxicity data.

## Introduction

Patients with mediastinal lymphoma commonly are young with curable histologies. While radiotherapy is an effective treatment for mediastinal lymphoma, there has been reluctance to irradiate these patients given increased risk of radiation-associated late cardiac toxicity [1–3] and secondary cancers of the breast [4, 5] and lung tissue [6]. Strategies to reduce toxicity in these patients have included reducing treatment volumes (involved site/node radiation), lowering radiation dose, and improving radiation delivery. Several techniques have been used in the latter setting, including butterfly intensity modulated radiation therapy (IMRT) [7], deep inspiration breath hold (DIBH) [8], and proton therapy [9].

Proton therapy’s physical dose distribution, which is associated with a steep dose fall-off beyond the Bragg peak, makes it an attractive radiation technique in patients with mediastinal lymphoma. However, there are unique treatment planning considerations that arise with proton therapy. Mostly, pencil beam analytical (PBA) dose algorithms have been used to calculate dose distributions, although PBAs may not calculate proton dose in lung tumors accurately given the heterogeneous tissue interfaces that proton beams must traverse [10, 11]. In addition, there is compelling theoretical and laboratory evidence indicating that within the Bragg peak, proton linear energy transfer (LET) and, therefore, relative biological effectiveness (RBE) varies [12, 13], although a constant clinical RBE of 1.1 is currently used for proton therapy planning and outcome assessment. In sites such as the mediastinum, uncertainties in the proton biological dose distribution (RBE × physical dose) arise from both the dose calculation algorithm and uncertainties associated with proton RBE. In our clinic, anterior or anterior oblique proton beams are typically used for mediastinum lymphoma treatment planning. There is a possibility that cardiac structures that lie at the distal end of these beams may be exposed to high linear energy transfer protons with an RBE greater than 1.1.

Numerous comparative dosimetric studies have compared proton and photon-based techniques for mediastinal lymphoma [14], although most, if not all, of these were performed using PBA and a constant clinical RBE of 1.1. Commercial Monte Carlo-based (MC) dose calculation algorithms have recently become available for clinical use. MC, which is regarded as the gold standard for physical dose calculations, also has the potential to incorporate variable RBE (vRBE) models that account for spatial variations in proton kinetic energy and LET within the Bragg peak. We evaluated the role of MC dose algorithms for proton treatment planning in the mediastinum and compared dosimetry between photons and proton plans that had been optimized and calculated with MC. A secondary goal was to explore the potential impact of vRBE on cardiac doses relative to ^60^Co γ-rays and MV x-rays. We strived to accomplish these goals by performing (i) dosimetric comparisons between proton PBA and MC based plans, (ii) dosimetric comparisons between photon and proton MC plans on DIBH and free breathing (FB) scans, and (iii) dosimetric comparison of cardiac structure doses for biological dose distributions [(physical dose × vRBE) vs (physical dose × 1.1)] for DIBH and FB proton plans and the photon DIBH plans (RBE = 1.0). Note, photon plans were only constructed on DIBH scans given that prior studies [15] have suggested that IMRT in DIBH, proton therapy in FB, and proton therapy in DIBH each significantly reduced estimated late effects and life-years lost compared to IMRT in FB. Therefore, we wanted to use to “best” photon plan as comparison to proton plans. Both protons in DIBH and FB were used as comparisons given that at many proton centers, including ours, DIBH may not be in routine use given lack of volumetric image guidance (e.g. cone-beam CT) and patients may instead be treated using FB.

## Materials and methods

### Treatment simulation and contours

After obtaining approval from the University of Washington Institutional Review Board, we retrospectively reviewed 8 consecutive mediastinal lymphoma patients that were simulated between January 1, 2015 and May 1, 2017 at the SCCA Proton Therapy Center or University of Washington. All patients underwent 4D free breathing (FB), DIBH, and a helical contrast CT simulation scans. Patients were simulated with a thermoplastic mask with gentle neck extension and arms down or akimbo (if the axilla was treated). The Active Breathing Coordinator system (Elekta, Stockholm, Sweden) was used for DIBH. A single radiation oncologist with expertise in lymphoma (YDT) contoured all cases using the involved site technique [16, 17] and retrospectively contoured cardiac substructures using a published contouring atlas [18]. One set of the cardiac contours were reviewed with a radiologist with expertise in cardiac imaging (GK) and feedback was incorporated before contours were finalized. For the FB 4D CT scan, internal target volumes (ITV) were drawn on the average intensity projection (AVE-IP) images and edited using the maximum intensity projection (MIP) and cine images. The CTVs were drawn on DIBH images. The CTV/ITV to PTV margin ranged from 5 to 7 mm across patients but was constant for comparative plans for a single patient.

Mediastinal lymphoma patients included those with classical Hodgkin lymphoma (*n* = 5), primary mediastinal B-cell lymphoma (*n* = 2), and grey zone lymphoma (*n* = 1). Median age at radiation CT simulation was 34 years (range, 18–38). Seven of 8 patients had mediastinal disease that extended below the left pulmonary artery (i.e. lower mediastinal involvement). Extent of disease is summarized in Table 1. Among the 8 patients that underwent simulation, 6 were treated with pencil-beam scanning (PBS) proton therapy in free breathing (Table 1), which was calculated using the pencil-beam algorithm. DIBH was not used with proton therapy given lack of volumetric image-guidance at our center. One patient refused radiotherapy after simulation (patient 5), and one patient (patient 6) was treated with 3D conformal photons in DIBH given that her disease was limited to the right neck and upper mediastinum and above the level of the left pulmonary artery. While prescription doses ranged from 20 to 45 Gy, for this dosimetric comparison study, the same prescription dose was used for all patients: 30 Gy in 15 fractions.

**Table 1:** Disease extent treated among 8 patients with mediastinal lymphoma

Patient	1	2	3	4	5	6	7	8
RT treatment plan delivered	Proton FB	Proton FB	Proton FB	Proton FB	Patient declined RT	3D conformal DIBH	Proton FB	Proton FB
Treatment fields for proton therapy planning	AP	RAO, LAO	RAO, LAO	RAO, LAO	AP	AP, RAO	AP	AP, RAO
Extent of disease involvement								
Neck	B			B	B	R	L	R
Axilla				L			L	
Upper mediastinum*	X	X	X	X	X	X	X	X
Mid-mediastinum**		X	X	X	X		X	X
Lower mediastinum***		X	X		X			
Posterior mediastinum (behind heart)	X							
Hilum	L							
IMN	R							

Abbreviations: AP, anterior-posterior; B, both left and right; DIBH, deep inspiration breath hold; FB, free breathing; L, left; LAO, left anterior oblique; R, right; RAO, right anterior oblique

*Disease goes down to left pulmonary artery

**Disease extends below left pulmonary artery and to inferior aspect of aortic valve

***Disease extends below level of aortic valve

### Proton treatment planning and robustness analysis

PBS proton plans were created on the DIBH and free breathing scans. The free-breathing plans were planned on the CT obtained by averaging the ten phases from a 4D CT acquisition. The DIBH plans were performed on static CT obtained with the patient under breath-hold and monitored using the Automatic Breathing Coordinator™ device (Elekta Inc., Sweden). Proton plans were calculated in the RayStation treatment planning system (version 6) using a PBA-based dose algorithm (version 4.1) and MC-based dose algorithm (version 4.0). The beam model corresponds to the commissioned IBA Proteus Plus beam at Seattle Cancer Care Alliance Proton Therapy Center [19]. Anterior or anterior oblique beams within +/− 30 degree from vertical were used. Single field uniform dose optimization technique was used with at least two beams, and 2X volumetric repainting was performed for patients with excessive motion (> 1 cm) in the target area. The decision to use 2X volumetric repainting is based on results from the Monte Carlo study by Grassberger et al. [20]. In this study, the optimal number of repaintings was calculated to preserve target coverage based on tumor size and its motion. The subsequent dosimetric endpoints provided in the results section account for 2X volumetric repainting, wherever it is applicable. All 8 patients had PTV volumes extending <=7.5 cm to patient’s external contour. Thus, a 7.5 cm water equivalent thickness range shifter of acrylic material was inserted into the beam path to ensure adequate coverage of the target superficially. The lateral spot and energy layer spacing parameters were set to an automatic scale of 1 for most proton plans and reduced in 0.2 increments to below 1 only for plans where coverage could not be met. The spot spacing parameter for proton plans in the RayStation treatment planning system determines the lateral grid spacing for proton spot placement. A higher value increases the inter-spot distance. Similarly, the energy layer spacing parameter determines the longitudinal spacing between Bragg peaks. The optimal value of these two parameters must provide the optimizer sufficient flexibility during the optimization process without unduly increasing the times for plan optimization and treatment delivery. For this study, the default value of 1 was found to be suitable for both these parameters, as also suggested by Alshaiki et al. [21].

All nominal proton plans were optimized so that at least 99% of the CTV/ITV achieved 99% of prescribed dose (CTV or ITV V_99% RX_ > 99%). Similarly, the nominal plans also ensured that at least 95% of the PTV achieved 99% of the prescribed dose (PTV V_99% RX_ > 95%). After achieving the desired target dose levels, the plans were further optimized to reduce OAR doses (in order of highest priority) to the heart, breast tissue (for females), and lungs.

The nominal plans were perturbed for set up and range errors, including under and over ranging of 3% and isocenter shifts of +/− 3 mm in the superior/inferior, anterior/posterior, and left/right directions. Our institution criterion is to ensure that at least 95% volume of CTV/ITV is covered by 95% of prescription dose in perturbed conditions (CTV or ITV V_95% RX_ > 95%). For this study, all the perturbed plans achieved institution criteria with minimum CTV or ITV V_95%RX_ of 97.9%.

To evaluate the impact of MC dose algorithm, original proton plans that were optimized and calculated with PBA algorithm (PBPB), were retrospectively re-calculated with MC dose algorithm (PBMC). The MC algorithm, as implemented in the RayStation planning system, has been shown to be highly accurate for dose calculation in heterogeneous media [22–26] as encountered in the treatment of mediastinal lymphoma. The clinical implementation of MC for treatment planning at the SCCA proton therapy center has been published [27]. New plans were also created using MC for plan optimization and final dose calculation (MCMC). These plans were done with a constant RBE of 1.1 and on free breathing scans (FB), therefore resulting in three different study arms: i.e., FB PBPB, FB PBMC, and FB MCMC. MC was also used to optimize and calculate final proton dose on DIBH scans. These plans also used a constant RBE of 1.1 and are therefore referred in Tables 2 and 3 as DIBH MCMC.

**Table 2:** Summary of target volume coverage, heterogeneity index, and dose to organs at risk for proton plans calculated with pencil beam algorithm (PBA), recalculation of PBA plan using Monte Carlo (MC), re-optimized plan using MC and fixed RBE, and re-optimized plan using MC with variable RBE among 8 mediastinal lymphoma patients with free-breathing CT simulation scans (left 4 columns). Comparison plans on deep inspiration breath hold (DIBH) scans with optimized PBS plan using MC versus photon techniques. Median volume or dose with interquartile range (IQR) in parentheses. Pair-wise dose differences were compared using a Wilcoxon signed-rank test. Significant *p*-values (< 0.05) are bolded for emphasis

Median (IQR)	FB PBPB cRBE	FB PBMC cRBE	FB MCMC cRBE	FB MCMC vRBE	DIBH MCMC cRBE	DIBH IMRT
*ITV or CTV target coverage*						
V99*	100% (100–100%)	80.6% (66.2–91.4%)	99.9% (99.6–100%)	98.7% (69.8–99.4%)	99.7% (99.6–99.9%)	99.3% (99–99.8%)
V95	100% (100–100%)	98.4% (96.7–99.3%)	100% (100–100%)	100% (99.8–100%)	100% (99.9–100%)	99.9% (99.8–100.0%)
Global dose max	107.6% (106.1–108.1)	108.1% (105.5–109.1)	109.2% (107.9–109.5)	112.5% (110.5–114.4)	109.7% (109.4–109.9)	112.3% (111.2–112.7%)
Homogeneity index (D95/D5)	0.97 (0.95–0.97)	0.94 (0.93–0.96)	0.97 (0.97–0.97)	0.95 (0.95–0.96)	0.96 (0.96–0.97)	0.96 (0.95–0.96)
*Dose to normal structures*						
Mean lung (Gy)	6.6 (4.4–9.2)	6.6 (4.6–9.2)	7.1 (5.0–8.9)	7.5 (5.7–9.0)	5.9 (4.8–7.8)	8.9 (8.1–11.7)
Lung V20	16.3% (9.7–21.4)	16.1% (9.6–21.3)	16.8% (10.7–20.1)	17.1% (13.8–20.5)	12.3% (9.8–16.5%)	17.6% (10.6–24.8)
Lung V5	30.5% (22.3–45.4)	31.4% (23.3–47.2)	33.4% (25.4–43.9)	34.9% (26.4–44.5)	31.0% (24.2–38.4%)	53.3% (45.1–61.6)
Mean heart (Gy)	9.3 (7.6–12.2)	9.1 (7.4–11.8)	9.9 (7.7–12.0)	10.2 (8.7–12.0)	8.6 (6.5–10.9)	10.4 (9.0–15.1)
Mean left breast (Gy)	2.0 (0.5–4.2)	2.1 (0.6–4.3)	2.2 (0.6–4.8)	2.0 (0.6–4.5)	2.1 (0.6–4.0)	4.1 (1.2–7.5)
Mean right breast (Gy)	1.2 (0.3–2.6)	1.3 (0.4–2.7)	1.4 (0.6–2.9)	1.7 (0.6–2.9)	1.4 (0.9–3.3)	2.9 (1.9–5.6)
Max spinal cord (Gy)	13.8 (13.0–17.2)	16.5 (14.4–20.0)	17.9 (15.4–20.9)	19.0 (16.6–21.5)	19.7 (15.0–21.0)	30.4 (27.0–31.7)
Max esophagus (Gy)	31.5 (31.3–32.3)	31.0 (30.7–31.3)	32.1 (31.1–32.5)	31.9 (31.3–32.8)	32.0 (31.7–32.3)	31.7 (31.6–32.5)
*P*-value	FB PBPB cRBE vs FB PBMC cRBE	FB PBMC cRBE vs FB MCMC cRBE		FB PBPB cRBE vs FB MCMC cRBE	FB MCMC cRBE vs DIBH photon	DIBH MCMC cRBE vs DIBH photon
*ITV or CTV target coverage*						
V99*	**0.012**	**0.018**		0.093	0.12	0.16
V95	**0.012**	**0.012**		0.20	0.075	0.36
Global dose max	0.48	0.16		0.06	**0.012**	**0.012**
Homogeneity index (D95/D5)	**0.018**	**0.012**		0.28	0.25	0.20
*Dose to normal structures*						
Average lung (Gy)	0.08	0.21		0.16	**0.012**	**0.012**
Lung V20	0.29	0.33		0.33	0.26	0.16
Lung V5	**0.012**	0.33		0.05	**0.012**	**0.012**
Average heart (Gy)	**0.012**	0.093		1.00	0.16	**0.025**
Average left breast (Gy)	0.35	0.12		**0.046**	**0.028**	**0.028**
Average right breast (Gy)	**0.028**	**0.043**		**0.028**	**0.028**	**0.046**
Max spinal cord (Gy)	0.05	0.33		**0.017**	**0.012**	**0.017**
Max esophagus (Gy)	**0.012**	**0.011**		0.58	0.78	1.00

^*^Volume of ITV or CTV covered by 99% isodose line

*Abbreviations*: *FB* Free breathing; DIBH, deep inspiration breath hold; cRBE, constant RBE; MCMC, plan optimized and calculated with Monte Carlo algorithm; PBMC, plan optimized with pencil-beam algorithm and re-calculated with Monte Carlo algorithm; PBPB, plan optimized and calculated with pencil-beam algorithm; vRBE, variable RBE

**Table 3:** Comparison of dose (mean and maximum) to cardiac substructures between proton plan with free breathing (FB), protons with deep inspiration breath hold (DIBH), and photons with DIBH. Proton plans were optimized with Monte Carlo dose algorithm and calculated with a variable relative biological effectiveness (RBE)

	Median, Gy (IQR)		
	FB MCMC vRBE	DIBH MCMC vRBE	DIBH photon
Mean left main coronary	23.9 (16.8–27.7)	22.4 (17.1–27.3)	23.0 (19.3–29.0)
Max left main coronary	26.5 (16.9–28.6)	26.8 (22.6–29.7)	29.5 (26.4–31.1)
Mean left anterior descending	12.4 (4.9–22.3)	13.7 (4.8–20.8)	12.4 (6.3–22.5)
Max left anterior descending	29.3 (19.7–31.0)	29.5 (21.9–31.3)	28.3 (23.9–32.1)
Mean left circumflex	9.5 (4.3–16.3)	8.0 (3.1–11.8)	10.8 (6.8–17.0)
Max left circumflex	29.1 (18.4–30.0)	27.3 (18.1–30.0)	29.9 (18.7–31.3)
Mean right coronary artery	23.8 (14.6–26.1)	20.8 (10.3–26.5)	23.8 (8.5–29.0)
Max right coronary artery	31.3 (30.8–31.8)	31.1 (30.3–31.9)	31.8 (30.5–32.1)
Mean left atrium	9.4 (4.5–18.3)	6.4 (3.4–12.0)	11.8 (7.7–16.8)
Max left atrium	31.3 (23.6–32.2)	31.8 (25.9–32.2)	31.8 (30.5–32.2)
Mean left ventricle	5.7 (0.2–9.0)	4.2 (0.3–7.0)	6.2 (4.1–10.8)
Max left ventricle	30.0 (8.7–32.3)	30.8 (10.7–32.3)	31.6 (22.2–32.4)
Mean right atrium	10.4 (8.1–19.0)	11.2 (5.5–18.1)	21.3 (6.1–26.1)
Max right atrium	31.9 (31.1–32.3)	32.0 (31.6–32.4)	32.4 (32.1–32.6)
Mean right ventricle	12.5 (6.6–14.1)	8.3 (6.0–15.7)	11.0 (8.0–15.7)
Max right ventricle	32.1 (31.6–32.7)	32.8 (32.4–33.3)	32.3 (31.9–33.2)
Mean aortic valve	11.6 (5.8–18.6)	9.3 (3.3–13.1)	12.5 (9.1–21.1)
Max aortic valve	26.7 (21.8–29.5)	23.9 (20.2–25.9)	28.7 (25.7–30.4)
Mean mitral valve	2.2 (0.1–5.8)	0.9 (0.1–2.1)	6.1 (3.5–9.7)
Max mitral valve	9.8 (0.5–23.1)	4.9 (0.4–19.8)	11.5 (6.3–24.3)
Mean pulmonic valve	27.4 (26.5–29.9)	28.3 (26.8–30.0)	29.6 (23.6–30.7)
Max pulmonic valve	30.8 (30.5–31.5)	30.9 (30.5–31.7)	32.1 (31.9–32.3)
Mean tricuspid valve	4.2 (1.9–6.9)	1.8 (0.4–8.1)	10.2 (2.4–20.9)
Max tricuspid valve	26.4 (19.5–28.8)	22.7 (3.2–30.1)	26.2 (5.7–32.1)

*Abbreviations*: FB, free breathing; DIBH, deep inspiration breath hold; cRBE, constant RBE; MCMC, plan optimized and calculated with Monte Carlo algorithm; PBMC, plan optimized with pencil-beam algorithm and re-calculated with Monte Carlo algorithm; PBPB, plan optimized and calculated with pencil-beam algorithm; vRBE, variable RBE

### Photon treatment planning

Comparative photon plans were generated on the DIBH scan in RayStation with the collapsed cone convolution superposition dose engine (version 3.4). Collapsed cone convolution algorithms accurately predict dose in inhomogeneous tissues such as the mediastinum [28, 29]. Implementation of this algorithm in the RayStation photon treatment planning system was found to be accurate in determining point doses in anthropomorphic thorax phantom with composite doses within +/− 1% [30].

The same DIBH CTV/PTV coverage and OAR dose constraints used for proton planning were used for photon dose optimization. Dynamic MLC form of IMRT was used with 6 MV photon beam energy. Five to seven beams were used for IMRT with the butterfly technique [7]. The photon plans are referred to as DIBH Photon in Tables 2 and 3.

### Evaluation of cardiac structure dosimetry with a vRBE model

To assess the potential impact of spatial variations in proton RBE within the Bragg peak (i.e., vRBE), we used a published model for DNA double-strand break (DSB) induction [31–34] implemented into a research version of RayStation (version 6R). As explained in the Appendix, the RBE for DSB induction is closely related to the RBE for cell survival [35, 36] for protons with LET up to ~ 15 keV/μm (kinetic energies > 2 MeV and a continuous slowing down approximation (CSDA) range in water > 0.07 mm). This LET range encompasses all regions of a pristine Bragg peak except those regions a millimeter or so beyond the tip of the Bragg peak (Additional file 1: Figure S1). The RBE-weighted dose, which is a product of RBE and physical dose, is hereafter referred to as biological dose or as dose. Biological dose distributions computed using the vRBE model are compared to biological dose distributions with a constant clinical RBE = 1.1 and to photon dose distributions (implicit constant RBE = 1.0).

### Statistical analysis

Absolute dose differences and dose-volume metrics across radiation plans were compared using Wilcoxon signed-rank test for non-parametric paired data. All tests were 2-tailed and *P* values < 0.05 were considered statistically significant.

## Results

### Proton treatment planning with Monte Carlo

**Fig. 1:**
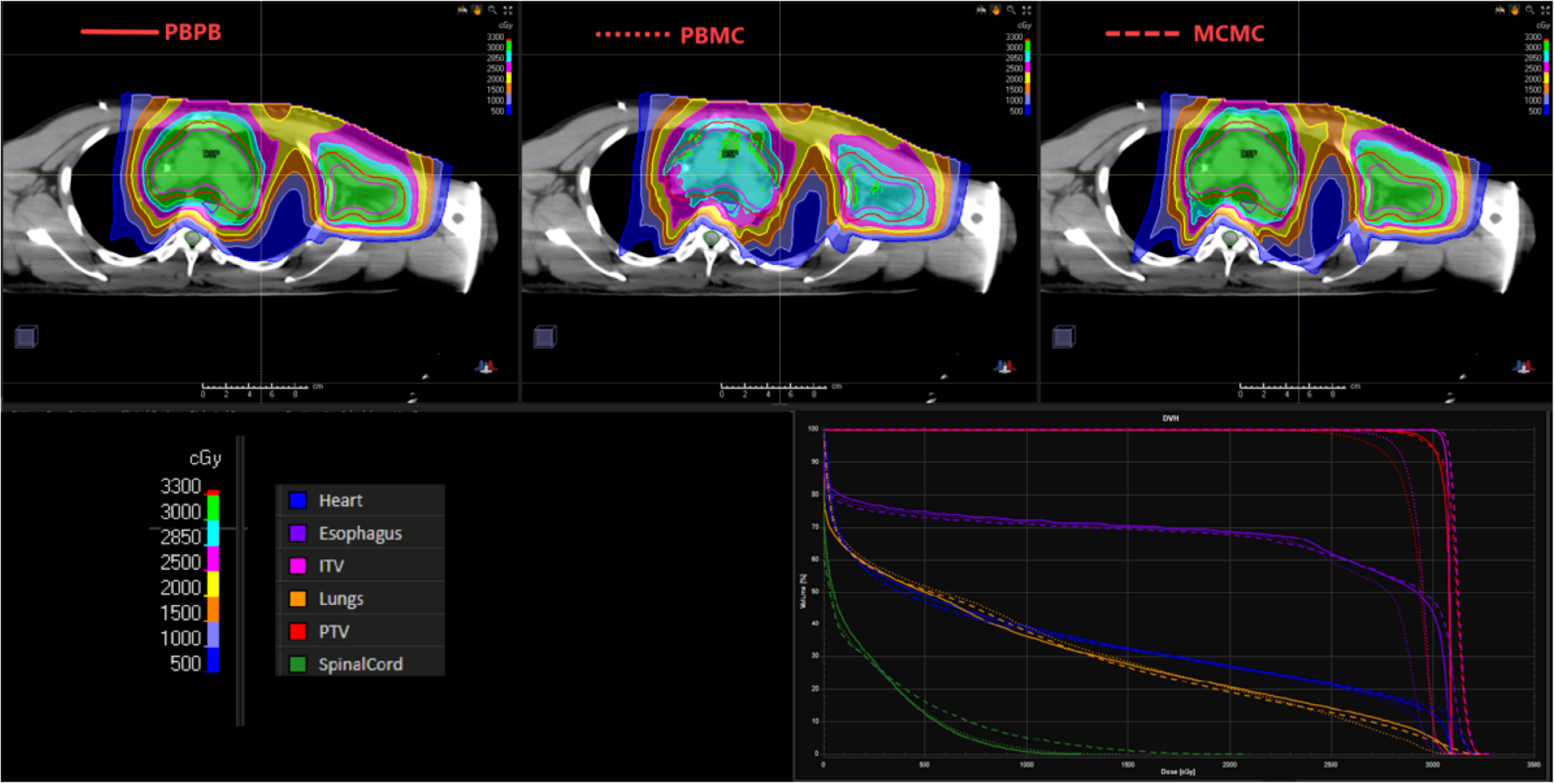
Axial images of a representative patient comparing dose distribution from pencil-beam analytical algorithm plan (PBA; PBPB), PBA plan recalculated with Monte Carlo (PBMC), and PBA plan re-optimized with Monte Carlo (MCMC). Dose-volume histogram for ITV target coverage demonstrating how initial coverage in the PBPB plan (solid line) is lost in the PBMC plan (dotted line). Target coverage was recovered with MCMC plan (dashed line)

**Fig. 2:**
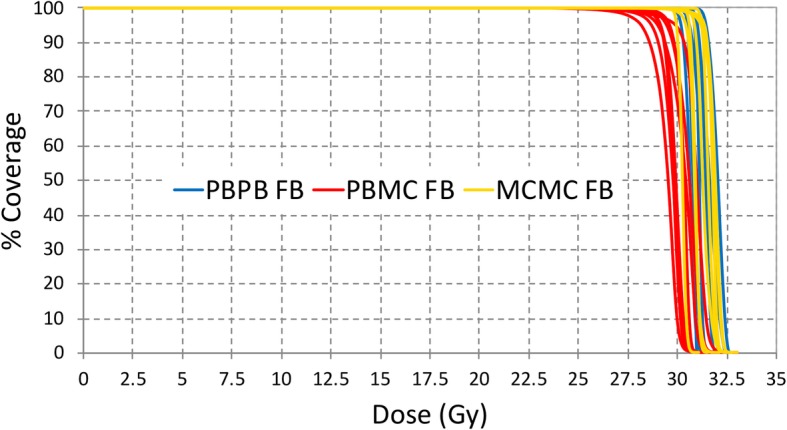
Dose-volume histograms for each patient’s ITV coverage, based on PBS plans generated with the pencil beam algorithm (PBPB FB), pencil beam algorithm plan recalculated with Monte Carlo (PBMC FB), and Monte Carlo optimized plan (MCMC FB). All plans were generated on the free breathing (FB) scan

Re-calculation of FB PBPB plans with MC (FB PBMC plan) demonstrated significant under coverage of the ITV V99 (*p* = 0.012) and V95 (p = 0.012; Table 2, Figs. 1 and 2). The average and median reductions for ITV V99 were 24.1% and 19.4%, respectively. The coverage loss was less for ITV V95, with average and median reduction of 2.5% and 1.6%, respectively. Compared to FB PBPB plans, FB PBMC plans showed greater heterogeneity in target dose levels with lower median homogeneity index (D_95%_/D_5%_): 0.94 versus 0.97. There was also increase in spinal cord dose maximum (*p* = .05) with average and median increase of 2 Gy and 2.7 Gy, respectively.

ITV coverage in FB PBMC plans was recovered by re-optimizing and calculating plans with MC (FB MCMC plan), with similar global dose maximum and homogeneity index as the original PBPB plans. Dose to the heart and lungs were not significantly different between FB PBPB and FB MCMC plans. Statistically, but not clinically, significant different doses to the mean right breast and maximum esophageal dose were observed (Table 2). There was also increase in spinal cord maximum dose for FB MCMC plans over FB PBPB plans, with average and median increase of 3.1 Gy and 4.1 Gy, respectively.

### Comparison of photon versus proton plans with constant RBE

**Fig. 3:**
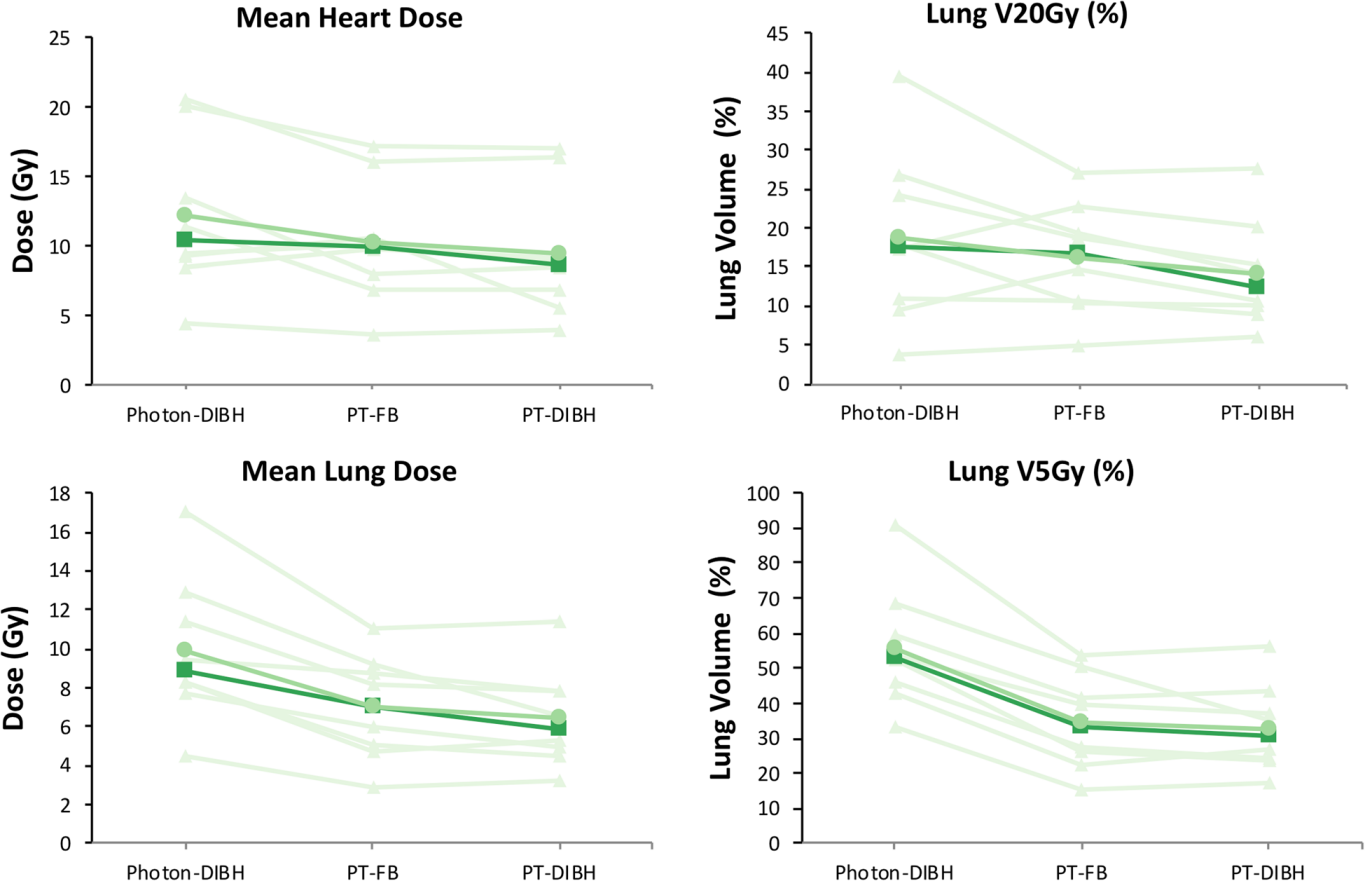
Paired scatter plot of dose to lung (mean, V5, V20) and heart (mean) for each patient from photon DIBH, proton free breathing, and proton DIBH plans. For proton plans, MC was used for optimization and final dose calculation. Individual patient data is plotted in triangles and light green. Mean dose difference is represented by circles and medium green. Median dose difference is represented by squares and dark green

Pairwise comparison for both FB MCMC and DIBH MCMC proton plans was performed to DIBH photon plans. Proton plans had similar target coverage and homogeneity index as photon plans. Both DIBH and FB proton plans had lower average mean lung dose by 3 Gy and 1.4 Gy, respectively (Table 2; Fig. 3). There was marked reduction in V5Gy lung volume by 20% for proton plans, likely secondary to fewer beams used for proton plans and lack of exit radiation dose.

Proton DIBH plans also showed statistically significant reduction in mean heart dose over photon plans with median and average reduction of 1.8 Gy and 3.1 Gy, respectively (Table 2; Fig. 3). Proton plans also had reduced spinal cord maximum dose with average decrease of >9Gy for both DIBH and FB plans. The spinal cord dose differences could be attributed to beam angles that are required for plans. Proton plans can achieve desired target coverage levels with only anterior beams thus minimizing spinal cord dose. Photons plans, on the other hand, also used posterior beams that traversed through spinal cord thus increasing spinal cord dose.

### Comparison of photon and vRBE proton dose to cardiac substructures

Dose to cardiac substructures were compared between DIBH photons and MC-optimized proton plans with and without DIBH. Given that proton plans use anterior weighted beams that could range into the heart (i.e., high LET protons with an RBE > 1.1), we evaluated dose to cardiac substructures using a vRBE model for the endpoint of DSB induction. The RBE for DSB induction is close to a linear function of proton LET up to about 15 keV/μm (Additional file 1: Figure S2) and is closely related to the RBE for cell survival [35, 36].

**Fig. 4:**
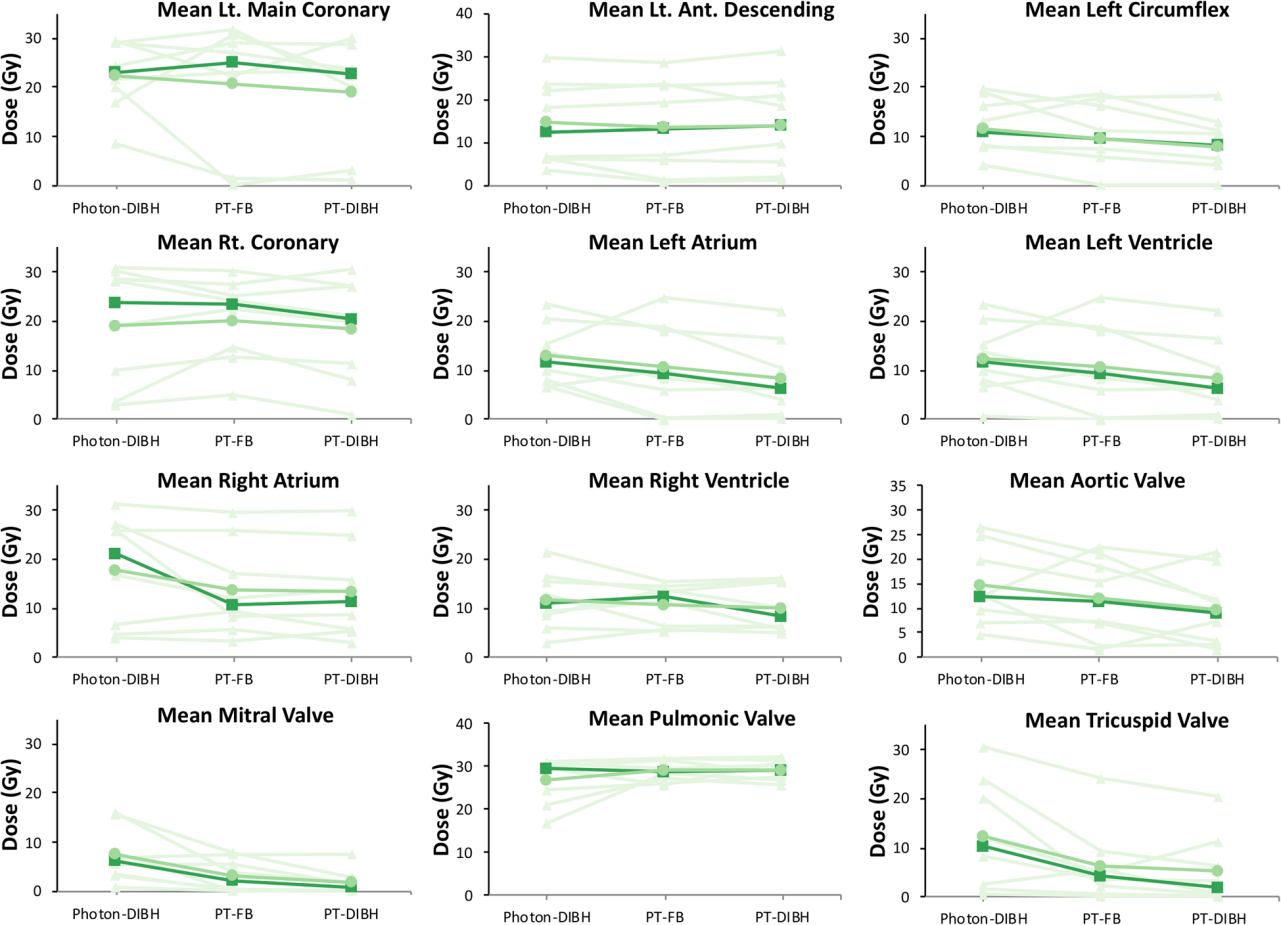
Paired scatter plot of mean dose to cardiac substructures for each patient from photon-DIBH, proton free breathing, and proton DIBH plans. For proton plans, MC was used for physical dose optimization and vRBE was applied for final dose calculation. Individual patient data is plotted in triangles and light green. Mean dose difference is represented by circles and medium green. Median dose difference is represented by squares and dark green

The relative benefit of proton therapy and DIBH on mean dose to cardiac substructures varied across patients (Fig. 4). The potential for an increase in RBE at the end of range was not found to be large enough to offset the dosimetric advantages of proton therapy (Table 3). Dose to cardiac substructures with proton therapy were effectively the same using a constant clinical RBE = 1.1 and with the vRBE model in our mediastinal patient population (results not shown).

## Discussion

To our knowledge, this is the first study evaluating the impact of MC dose algorithm and vRBE on proton dosimetry among mediastinal lymphoma patients. Although our study is limited by small patient numbers, MC dosimetry revealed reduced target coverage under PBA-based planning and increased dose heterogeneity, consistent with findings in lung cancer patients [10, 11]. Our study also revealed that dosimetric endpoints could be maintained if MC planning is used for both initial plan optimization and final dose calculation. These findings highlight the limitations of the PBA dose calculation algorithm to calculate dose across heterogeneous tissue interfaces (e.g. soft tissue, bone, lung) and suggest that MC dose calculation algorithm should routinely be used for proton treatment planning for patients with mediastinal lymphoma. Despite under coverage of the CTV/ITV, which in the worst-case scenario, 90% of the prescription dose covered 100% of the volume, none of the 6 patients treated with the nominal proton plans calculated with PBA developed recurrence at a median follow-up of 28.4 months from proton RT start, though our numbers are small.

Although this study is performed for pencil beam scanning proton therapy, the findings may still apply to passive scatter or uniform scanning proton techniques. The underlying analytical pencil beam dose calculation algorithm [37] used for proton pencil beam planning is the same for uniform scanning and passive scattering. For plans with similar anatomy and beam configuration, PBA plans may show similar limitations for dose calculations for these modalities.

The dosimetric superiority of proton over photon plans has been established [14], although prior comparative dosimetry studies used PBA, which may inaccurately estimate target volume coverage. Because of under-coverage of the target volume with PBA, re-optimization of plans using MC to improve target coverage was associated with slightly higher dose to nearby organs at risk. There has been recent interest in evaluating the impact of DIBH to photon or proton techniques [38–40], with the hypothesis that in certain subsets of patients, photons with DIBH may provide similar cardiac and/or lung sparing compared to protons with free breathing. Within our cohort, mean lung dose, lung V5Gy, spinal cord, and mean breast dose were lower with proton therapy free breathing compared with photon DIBH. Proton therapy with DIBH was also associated with lower mean heart dose and V20Gy compared with photon DIBH. Whether differences in mean dose to the breast, heart, and lung, which ranged in magnitude from 2 to 5 Gy, is clinically meaningful depends in part on the patient’s age [41], sex [41], prior treatments [3, 42], baseline co-morbidities [43], and family history [44], which can each modulate the risk of late toxicity from radiotherapy. For example, while there is a linear, no threshold relationship between dose to the breast tissue and risk of secondary cancer in Hodgkin lymphoma survivors [45], the relative risk is higher for a young (< 35 year old) female patient with intact ovarian function compared with a female in her fourth decade of life, whose fertility lifespan is more limited [41]. Therefore, any sparing of breast tissue from radiation will be more clinically meaningful in a young female patient. Age is only one dimension of risk; on top of this, other risk factors, as mentioned earlier, increase a patient’s baseline risk, and with it, the absolute excess risk of radiation-associated secondary cancer. For this reason, providing an objective cut off of “acceptable” dose differences (e.g. between photons and protons) is challenging given various other clinical and treatment factors that also modulate the risk of late radiation toxicity.

Addition of DIBH to proton therapy did not improve mean heart or breast dose, but as expected, DIBH improved lung metrics. Our findings are consistent with a larger cohort of 21 mediastinal lymphoma patients with lower mediastinal involvement, in which addition of DIBH did not impact mean heart dose with IMRT or proton therapy. Proton therapy had similar or lower dose to the heart, lung, and breast tissue [46]. In contrast, in the largest study to date from the University of Copenhagen, life years lost (LYL) attributable to late effects after radiotherapy for mediastinal Hodgkin lymphoma was calculated based on normal tissue dose generated with IMRT and proton plans, with and without DIBH [15]. Compared to IMRT-FB, proton therapy and IMRT-DIBH was associated with significantly lower LYL, but no difference was seen between proton therapy-FB and IMRT-DIBH. However, extent of mediastinal disease was not reported, which may be an important factor in which mediastinal lymphoma patients benefit from DIBH [8]. Recent consensus recommendations from ILROG highlight which mediastinal patients may benefit from proton therapy, including those with mediastinal disease that extends below the origin of the left main coronary artery [47].

Given that primarily anterior beams are used for our proton plans and concerns that the RBE at the proton beam’s end of range may exceed the currently used clinical RBE = 1.1, we also explored the biological dose to cardiac substructures using a published vRBE model for the endpoint of DSB induction. Our analysis of representative mediastinal patient plans indicates that, despite the potential for very large end-of-track RBE effects biological dose in cardiac substructures is not substantially increased. Biological dose was estimated using a single model; currently, no consensus exists on the most appropriate vRBE model to calculate biological dose.

Of note, although the RBE for DSB induction is one of the most biological significant forms of initial molecular damage and is closely related to cell survival (see Appendix), clinical endpoints such as local tumor control and normal tissue complications may not exhibit the same general trends in vRBE as molecular or cellular surrogates. On the other hand, vRBE models developed for the endpoint of reproductive cell survival have been in routine clinical use for high LET carbon ion therapy for some time with little or no evidence of unexpected normal-tissue damage or compromised local tumor control [48–51]. On the relevant spatial scales (few mm), corrections for vRBE modeling in carbon ion therapy are much larger than vRBE corrections in proton therapy (i.e., on the order of 3–5 compared to on the order of 1.0 to 1.4). Also, the same general molecular and cellular RBE mechanisms of action are largely believed the same for protons and carbon ions in vitro and in vivo [36, 52]. Although distal to the Bragg peak, RBE for cell survival is larger than the RBE for DSB and has the potential to become as large as 2–3.7 (blue shaded region in Additional file 1: Figure S1), the impact of differences in RBE estimates (DSB versus cellular survival) on biological dose is minimal as the proton physical dose is rapidly decreasing over these few millimeters beyond the Bragg peak.

Proton therapy was associated with lower dose to the left ventricle, aortic valve, mitral valve, and tricuspid valve, although the clinical significance of these differences is not clear. Risk of valvular heart disease (VHD) after cardiac irradiation is non-linearly related to dose to the affected valve, in which risk of VHD increases by only 2.5% per Gy with valve doses <=30 Gy [53]. As most patients within our cohort were treated with doses <=30 Gy (i.e. within the shallowest slope of the dose-response relationship), decrease in valvular dose may be associated with minimal changes in VHD risk. Mean left ventricle dose was relatively low among our cohort (range, 4.2–6.2 Gy across photon and proton plans), and while protons was associated with significantly lower dose, the clinical impact may be very small, if any, as the risk of heart failure is non-linearly related to mean left ventricular dose [54].

In conclusion, MC-based dosimetry revealed reduced target coverage under PBA-based planning. Proton plans optimized with MC dose algorithm confirmed modest sparing of normal tissue over photon techniques with DIBH, although the relative benefit varies between patients. MC-based dose algorithms should be used in proton treatment planning for patients with mediastinal lymphoma. Our preliminary study suggests that end of range RBE effects do not significantly impact biological dose in cardiac substructures for mediastinal targets, although these dosimetric estimates will require validation with late toxicity data.

### Supplementary information

**Additional file 1: Figure S1.** RBE for DSB induction (*RBE*_*DSB*_) and the low dose [compared to (α/β)_R_] RBE for cell survival (*RBE*_*LD*_). *Left Panel:* RBE as a function of proton kinetic energy. A (and grey shaded region) denotes the approximate range of proton energies (“energy layers”) incident on patient (~ 90 to 225 MeV). *Right Panel:* RBE as a function of the continuous slowing down approximation (CSDA) range for a monoenergetic proton in water. Filled green squares denote estimates of RBE DSB from track structure simulations [64]. Filled red squares [(α/β)_R_ = 10 Gy] and yellow triangles [(α/β)_R_ = 2 Gy] denote LEM IV estimates of the RBE for cell survival after a 1.8 Gy absorbed dose (LEM IV data adapted from [36]). Solid black lines in the left and right panel are estimates of RBEDSB from the MCDS [31, 33]. Dashed lines are computed using Eq. (1) with (α/β)_R_ = 1 Gy, 5 Gy and 10 Gy and an effective cell diameter of 4 μm. At the proton end of range (left panel, blue shaded region), the RBE for the last-traversed-cell may be as large as 2 to 3.7. However, the RBE for most cells near the tip of a pristine Bragg peak (red shaded regions) is likely to be much closer to 1.1 (~ 1.05 to 1.25). Distal to a pristine Bragg peak, *RBE*_*DSB*_ and the RBE for cell survival rapidly rises to values that may approach 2.0 to 3.7 (blue shaded region in left panel). **Figure S2.** Proton RBE as a function of linear energy transfer (LET). Solid black line: MCDS estimate of *RBE*_*DSB*_ [31, 33]. Dashed lines: RMF model [35, 36] with (α/β)_R_ = 1 Gy, 5 Gy and 10 Gy (cell diameter = 4 μm). Red filled circles: LEM IV model estimate of the RBE for cell survival with (α/β)_R_ = 2 Gy and 10 Gy [36]. Yellow triangles: Microdosimetric-Kinetic (MK) model estimate of the RBE for cell survival with (α/β)_R_ = 2 Gy and 10 Gy [36]. Filled cyan squares: Wedenberg et al. [63] model of the RBE (1.8 Gy) for cell survival with (α/β)_R_ = 2 Gy and 10 Gy. Filled stars: McNamara et al. [63] model of the RBE (1.8 Gy) for cell survival with (α/β)_R_ = 2 Gy and 10 Gy. Blue shaded region corresponds to an RBE slope in the range from + 0.03 to + 0.09 per keV/μm.

## Data Availability

The datasets used and/or analyzed during the current study are available from the corresponding author on reasonable request.

## Appendix vRBE model for DNA double Strand break (DSB) induction and reproductive cell survival

The induction of DSB by ionizing radiation is widely viewed as one of the most critical, initial molecular insults. The DSB formed by ionizing radiation give rise to both lethal and non-lethal chromosome aberrations as well as small-scale (point) mutations. DSB are the only major category of DNA damage that tends to increase with increasing proton LET [33, 34]. Other categories of DNA damage, such as single strand breaks (SSBs) and clusters of multiple base lesions, all tend to decrease in number with increasing particle LET [33, 34]. Although the vast majority of DSB are correctly rejoined (less than ~ 2–3% are incorrectly rejoined), general trends in the RBE for DSB induction (*RBE*_*DSB*_) are closely aligned with and predictive of the RBE for reproductive cell survival [36]. In the mechanism-inspired Repair-Misrepair-Fixation (RMF) model [35], *RBE*_*DSB*_ is related to the RBE for cell survival by [36]:

A.1 $$ RB{E}_{LD}= RB{E}_{DSB}\left(1+\frac{2{\overline{z}}_F RB{E}_{DSB}}{{\left(\alpha /\beta \right)}_R}\right)\ge RB{E}_{DSB} $$

and

A.2 $$ RB{E}_{HD}= RB{E}_{DSB}\ge 1. $$

Here, *RBE*_*LD*_ is the RBE for cell survival in the limit as the fraction size *D* becomes small compared to (α/β)_R_, and *RBE*_*HD*_ is the RBE for cell survival in the limit as *D* becomes larger compared to (α/β)_R_. For any fraction size D, the RBE for cell survival must fall within the range *RBE*_*HD*_ = *RBE*_*DSB*_ < RBE < *RBE*_*LD*_. In Eqs. (A.1) and (A.2), (α/β)_R_ determines the sensitivity of a cell, tissue or tumor target to MV x-rays (i.e., the *reference radiation*) and ${\overline{z}}_F$ is the frequency-mean specific energy [56] in units of Gy for a particle type and kinetic energy of interest. As a first approximation, such as when the range of a proton is large compared to the diameter *d* of the cell nucleus (~ few μm), ${\overline{z}}_F\cong LET/\rho {d}^2$ and *ρ* is the material density (g cm^− 3^) of the cell nucleus.

For the important special case when $2{\overline{z}}_F RB{E}_{DSB}/{\left(\alpha /\beta \right)}_R<<1,$ the low dose RBE for cell survival [Eq. (A.1)] approaches *RBE*_*DSB*_; therefore, *RBE*_*LD*_ ≅ *RBE*_*HD*_ = *RBE*_*DSB*_. In this limit (*approximation*), the RBE for cell survival becomes independent of (α/β)_R_ and the fraction size *D*. As illustrated in the left panel of Additional file 1: Figure S1 for a representative human cell [*d* ~ 4 μm, (α/β)_R_ ~ 1 to 10 Gy], the RBE for cell survival is within a few percent (~ 1–10%) of *RBE*_*DSB*_ for protons with kinetic energies greater than about 10 MeV, which corresponds to an LET of 4.4 keV/μm and continuous slowing down (CSDA) range in water of 1.24 mm. It is only for those low energy (left panel, Additional file 1: Figure S1) very short range (< 1 mm) protons (right panel, Additional file 1: Figure S1) that the RBE for cell survival becomes substantially (> 10%) larger than *RBE*_*DSB*_.

Of note, a clinical RBE of 1.1 corresponds to an ~ 11 MeV proton with an LET of 4.2 keV/μm and CSDA range of 1.47 mm. Such a low-energy proton has a CSDA range comparable to or less than the dimensions of the volume elements (“voxel”) in a typical computed tomography (CT) scan or the dose grids used for treatment planning (~ 2–3 mm). As a first approximation, the tip of a pristine Bragg peak (red shaded region in Additional file 1: Figure S1) will occur in a voxel at the depth corresponding to a proton with a mean (*residual*) kinetic energy ~ 2–10 MeV (LET ~ 4.5 to 16.2 keV/μm). Distal to the Bragg peak as the kinetic energy falls below 1 MeV (blue shaded region in Additional file 1: Figure S1), the CSDA range of the proton becomes comparable to the diameter of a single cell (~ 10 μm) and the RBE may approach values as large as 2 to 3.7, depending on the value of (α/β)_R_. However, it’s important to recognize that far greater numbers of cells will experience an RBE ~ 1.15 + 0.1 (red shaded region) than to an RBE > 2. Near the tip of a clinical proton beam, the effective (dose- and cell-averaged) RBE for the endpoints of DSB induction and cell survival will be skewed much closer to an RBE ~ 1.1 to 1.2 rather than to an RBE > 2 (e.g., see Fig. 4 in [32]).

Additional file 1: Figure S2 shows a comparison of *RBE*_*DSB*_ from the Monte Carlo Damage Simulation (MCDS) to estimates of the RBE for cell survival from the RMF model [35, 36], the local effect model (LEM) version IV [57, 58], the microdosimetric-kinetic (MK) model [59–62], and the empirical Wedenberg et al. [63] and McNamara et al. [64] models. The LEM IV and the RMF model explicitly relate the RBE for cell survival to *RBE*_*DSB*_ (reviewed in [36]) whereas the MK, Wedenberg and McNamara models relate the RBE for cell survival to proton LET (or lineal energy) without any specific mechanism of action. All five models, as well as the *RBE*_*DSB*_ from the MCDS, exhibit a close-to-linear increase in proton RBE with increasing LET. With the exception of the McNamara et al. [64] model, the slope of the RBE is within the range from about 0.03 to 0.09 per keV/μm (blue shaded region in Additional file 1: Figure S2).
