# Supplementary figures and images for: Comparative photon and proton dosimetry for patients with mediastinal lymphoma in the era of Monte Carlo treatment planning and variable relative biological effectiveness
Source: Radiat Oncol. 2019 Dec 30;14:243. doi: 10.1186/s13014-019-1432-8 (PMC6937683; doi:10.1186/s13014-019-1432-8)

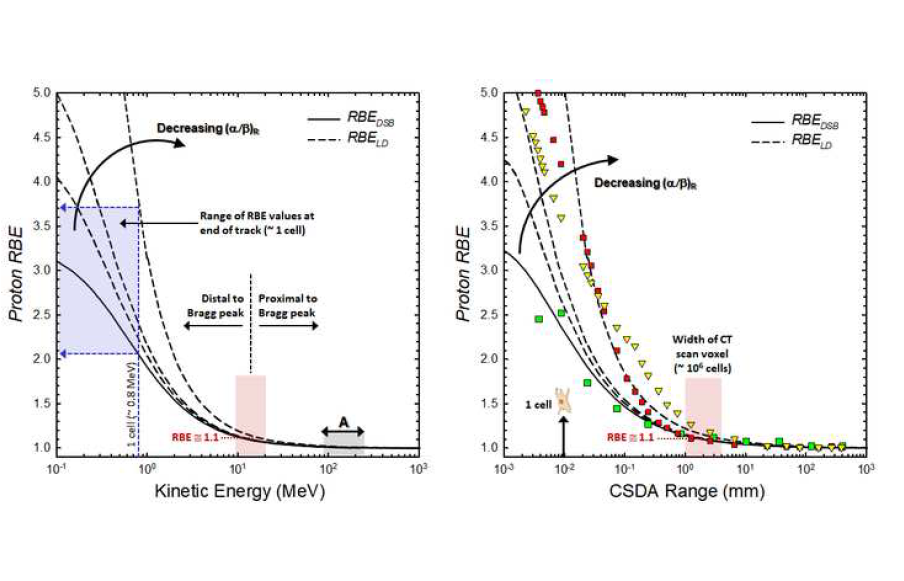

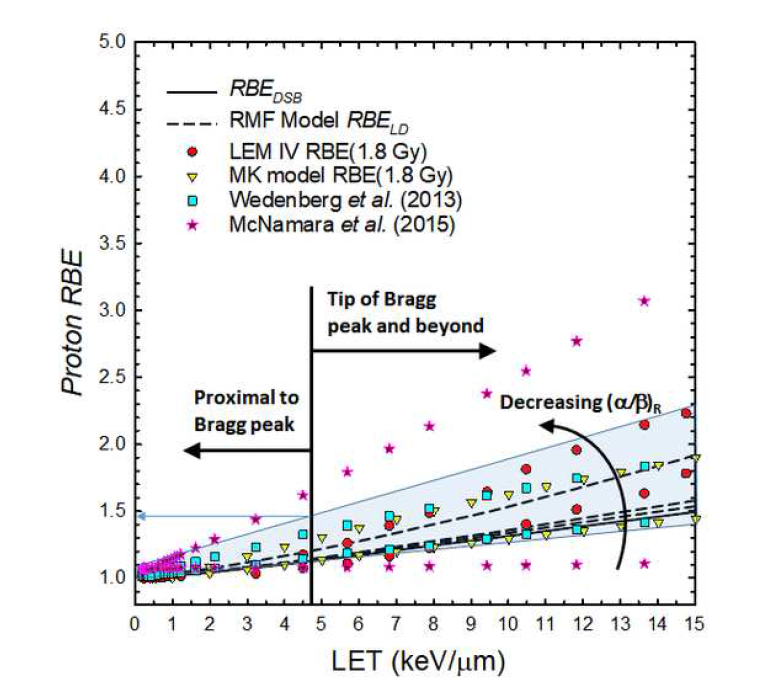

Supplement: Supplementary file 1 — Additional file 1: Figure S1. RBE for DSB induction (RBEDSB) and the low dose [compared to (α/β)R] RBE for cell survival (RBELD). Left Panel: RBE as a function of proton kinetic energy. A (and grey shaded region) denotes the approximate range of proton energies (“energy layers”) incident on patient (~ 90 to 225 MeV). Right Panel: RBE as a function of the continuous slowing down approximation (CSDA) range for a monoenergetic proton in water. Filled green squares denote estimates of RBE DSB from track structure simulations [64]. Filled red squares [(α/β)R = 10 Gy] and yellow triangles [(α/β)R = 2 Gy] denote LEM IV estimates of the RBE for cell survival after a 1.8 Gy absorbed dose (LEM IV data adapted from [36]). Solid black lines in the left and right panel are estimates of RBEDSB from the MCDS [31, 33]. Dashed lines are computed using Eq. (1) with (α/β)R = 1 Gy, 5 Gy and 10 Gy and an effective cell diameter of 4 μm. At the proton end of range (left panel, blue shaded region), the RBE for the last-traversed-cell may be as large as 2 to 3.7. However, the RBE for most cells near the tip of a pristine Bragg peak (red shaded regions) is likely to be much closer to 1.1 (~ 1.05 to 1.25). Distal to a pristine Bragg peak, RBEDSB and the RBE for cell survival rapidly rises to values that may approach 2.0 to 3.7 (blue shaded region in left panel). Figure S2. Proton RBE as a function of linear energy transfer (LET). Solid black line: MCDS estimate of RBEDSB [31, 33]. Dashed lines: RMF model [35, 36] with (α/β)R = 1 Gy, 5 Gy and 10 Gy (cell diameter = 4 μm). Red filled circles: LEM IV model estimate of the RBE for cell survival with (α/β)R = 2 Gy and 10 Gy [36]. Yellow triangles: Microdosimetric-Kinetic (MK) model estimate of the RBE for cell survival with (α/β)R = 2 Gy and 10 Gy [36]. Filled cyan squares: Wedenberg et al. [63] model of the RBE (1.8 Gy) for cell survival with (α/β)R = 2 Gy and 10 Gy. Filled stars: McNamara et al. [63] model of the RBE (1.8 Gy) for cell survival [file 13014_2019_1432_MOESM1_ESM.docx]
